# Oak (*Quercus frainetto* Ten.) Honeydew Honey—Approach to Screening of Volatile Organic Composition and Antioxidant Capacity (DPPH and FRAP Assay)

**DOI:** 10.3390/molecules15053744

**Published:** 2010-05-25

**Authors:** Igor Jerković, Zvonimir Marijanović

**Affiliations:** 1 Faculty of Chemistry and Technology, University of Split, N. Tesle 10/V, 21000 Split, Croatia; 2 Marko Marulić Polytechnic in Knin, P. Krešimira IV 30, 22300 Knin, Croatia

**Keywords:** *Quercus frainetto* Ten. honeydew honey, headspace solid-phase microextraction (HS-SPME), ultrasonic solvent extraction (USE), gas chromatography and mass spectrometry (GC and GC/MS), DPPH and FRAP assay

## Abstract

Two samples of oak honeydew honey were investigated. Headspace solid-phase microextraction (HS-SPME) combined with GC and GC/MS enabled identification of the most volatile organic headspace compounds being dominated by terpenes (mainly *cis*- and *trans*-linalool oxides). The volatile and less-volatile organic composition of the samples was obtained by ultrasonic assisted extraction (USE) with two solvents (1:2 (v/v) pentane -diethyl ether mixture and dichloromethane) followed by GC and GC/MS analysis. Shikimic pathway derivatives are of particular interest with respect to the botanical origin of honey and the most abundant was phenylacetic acid (up to 16.4%). Antiradical activity (DPPH assay) of the honeydew samples was 4.5 and 5.1 mmol TEAC/kg. Ultrasonic solvent extracts showed several dozen times higher antiradical capacity in comparison to the honeydew. Antioxidant capacity (FRAP assay) of honeydew samples was 4.8 and 16.1 mmol Fe^2+^/kg, while the solvent mixture extracts showed antioxidant activity of 374.5 and 955.9 Fe^2+^/kg, respectively, and the dichloromethane extracts 127.3 and 101.5 mmol Fe^2+^/kg.

## 1. Introduction

Unlike floral honeys, which derive from the nectar of flowering plants, honeydew honey is obtained by secretions of the living parts of plants or excretions onto them produced by sap-sucking insects. Honeydew honeys differ in chemical composition from nectar honeys [1] as well in the volatile composition and/or antioxidant activity. Screening of natural organic compounds that characterize these honey types is of particular interest because palynological analysis cannot be carried out [2]. *trans*-β-Methyl-γ-octalactone, a characteristic volatile compound of oak wood, is proposed as a chemical marker for the plant origin of oak honeydew honeys [2]. Other compounds such as aminoacetophenone and propylanisole can be considered characteristic of holm-oak honeydew honeys. 1-(2-Furanyl)-ethanone, butane-2,3-diol, 3-hydroxy-butan-2-one and 1-hydroxy-propan-2-one were suggested compounds for discrimination among nectar and honeydew honeys [1].

Antioxidant capacity of different honeys varies by floral source [3] as well by processing and storage conditions [4,5]. Components that were identified and/or quantified as honey antioxidants included phenolic compounds, ascorbic acid, the enzymes glucose oxidase, catalase, peroxidase and others. Additional research on single phenolic and other compounds in honey indicate that the antioxidant capacity is due to combination of a wide range of honey active compounds beyond phenolics [6]. Many different methods are appropriate for assessing the antioxidant activity (FRAP assay (ferric reducing antioxidant power), DPPH (1,1-diphenyl-2-picrylhydrazyl) method, ORAC (oxygen radical absorbance capacity), TEAC (Trolox equivalent antioxidant activity) and others) and in most cases it is necessary to use several tests to obtain good reliability [7]. Little information is available on the potential antioxidant activity of the honey ultrasonic solvent extracts. In our previous research [8] we reported the scavenging ability of the series of concentrations of the *Amorpha fruticosa* honey ultrasonic solvent extracts and the corresponding honey samples that was tested by a DPPH assay. Approximately 25 times lower concentration ranges (up to 2 g/L) of the extracts exhibited significantly higher free radical scavenging potential with respect to the honey samples.

*Quercus frainetto* Ten. belongs to the oak species. The growth process of oak fruits is extremely rich in cycles of 5-8 years. At the point of natural reduction of overproduction of fruits, the sweet sap from fruits appears. Fruit sap starts to flow over the cuticle of the fruit often with a foamy appearance. Therefore, the corresponding honeydew can also be produced without the mediation of plant sucking insects. Oak honeydew volatiles from Spain were previously identified by a microscale SDE apparatus after dichloromethane extraction [2] and *trans*-β-methyl-γ-octalactone, a characteristic volatile compound of oak wood, was proposed as a chemical marker. This compound is well known in winemaking, because it is responsible for the oak aroma of barrel-aged wines [9]. 

The scope of this research is to obtain new information of oak honeydew volatile organic composition by combined use of headspace solid-phase microextraction (HS-SPME) and ultrasonic solvent extraction (USE). In addition, the obtained USE extracts were tested by DPPH and FRAP assay for the first time in order to unlock their antiradical and antioxidant potential. DPPH and FRAP assay of the USE extracts were compared to the activity of the honeydew samples to obtain data of potential extra value of the solvent extracts, not just for organic analyses. 

## 2. Results and Discussion

The bees readily collect the plant sap on oak and *Quercus frainetto* Ten. is recognized as a specific source of honeydew in the region where the samples were collected. Besides sap-sucking insects that excreted exudates, sweet sap from oak fruits is noted. There were noticeably smaller amounts of honeydew elements in the sample sediments. Water content in the honeydew samples was 16.6% (sample I) and 16.2% (sample II). Electrical conductivity was 0.91 mS/cm (sample I) and 1.03 mS/cm (sample II).

### 2.1. Volatiles Isolated by Headspace Solid-Phase Microextraction

Headspace solid-phase microextraction (HS-SPME) combined with GC and GC/MS enabled identification of the most volatile organic headspace compounds in the samples. This approach for honey profiling is very important for obtaining reliable composition of the most volatile organic compounds. 

The most abundant compounds in the headspace of samples I and II were terpenes: *trans*-linalool oxide (18.1%; 13.8%), *cis*-linalool oxide (10.8%; 14.0%), hotrienol (4.4%; 9.7%) and epoxylinalool (4.4%; 2.0%). Linalool oxides and hotrienol were already found in oak honeydew [2]. Another abundant group of organic compounds were aliphatic acids: octanoic acid (8.5%; 9.4%), nonanoic acid (2.8%; 4.7%), decanoic acid (1.5%; 2.4%) and hexadecanoic acid (3.7%; 3.8%). No specific headspace marker compounds were found. Ubiquitous benzene derivatives in all honeys were also found: 2-phenylethanol (3.5%; 6.0%), phenylacetaldehyde (2.0%; 1.5%) and benzyl alcohol (2.4%; 4.8%). High concentrations of phenyacetaldehyde and 2-phenylethanol and lower quantity of benzaldehyde were found in oak honeydew from Spain [2]. Although furan derivatives were found particularly in the sample I (2-furancarboxaldehyde (0.7%), 2-furanmethanol (1.3%), 1-(2-furanyl)-ethanone (1.1%) and 5-methyl-2-furfural (8.4%)) their percentages in Table 1 are not reliable due to high polarity and low volatility.

**Table 1:** Oak honeydew organic headspace volatiles composition isolated by HS-SPME.

No.	Compound	RI	Area percentage (%)	
			sample I	sample II
1.	Pentan-1-ol^a^	< 900	0.5	0.9
2.	2-Methylbutan-1-ol	< 900	0.7	-
3.	2-Furancarboxaldehyde	< 900	2.0	-
4.	3-Methylbutanoic acid (Isovaleric acid)	< 900	0.8	-
5.	2-Furanmethanol	< 900	1.3	5.2
6.	1,3-Dimethylbenzene^**^	< 900	0.4	-
7.	1-(2-Furanyl)-ethanone	914	1.1	-
8.	5-Methylfurfural	969	8.4	3.5
9.	Hexanoic acid^a^	974	0.7	2.2
10.	2-Ethyl-1,3-dimethylbenzene^*^	1032	0.7	-
11.	2-Ethylhexan-1-ol^*^	1035	1.1	-
12.	Benzyl alcohol^a^	1037	2.4	4.8
13.	Phenylacetaldehyde^a^	1048	2.0	1.5
14.	*trans*-Linalool oxide (furan type)	1076	18.1	13.8
15.	Methyl 2-furoate	1084	-	2.8
16.	*cis*-Linalool oxide (furan type)	1091	10.8	14.0
17.	Hotrienol	1106	4.4	9.7
18.	2-Phenylethanol^a^	1116	3.5	6.0
19.	3,5,5-Trimethyl-cyclohex-2-en-1-one (α-Isophorone)	1124	0.6	-
20.	2-Ethylhexanoic acid	1140	2.9	-
21.	Neroloxide	1162	2.1	-
22.	Epoxylinalool	1178	4.4	2.0
23.	Octanoic acid^a^	1190	8.5	9.4
24.	Lilac alcohol (isomer I)	1208	0.8	-
25.	Lilac alcohol (isomer II)	1237	1.3	-
26.	Ethyl phenylacetate	1253	1.2	-
27.	Nonanoic acid^a^	1273	2.8	4.7
28.	Methyl cinnamate	1312	1.1	-
29.	Decanoic acid^a^	1370	1.5	2.4
30.	Hexadecanoic acid^a^	1963	3.7	3.8
Total identified			89.8%	86.7%

RI = retention indices on HP-5MS column; - = not identified; ^a^ – identification confirmed with reference compound; ^*^ - tentatively identified; ^**^ - correct isomer not identified.

### 2.2. Volatiles Isolated by Ultrasonic Solvent Extraction

**Figure 1:**
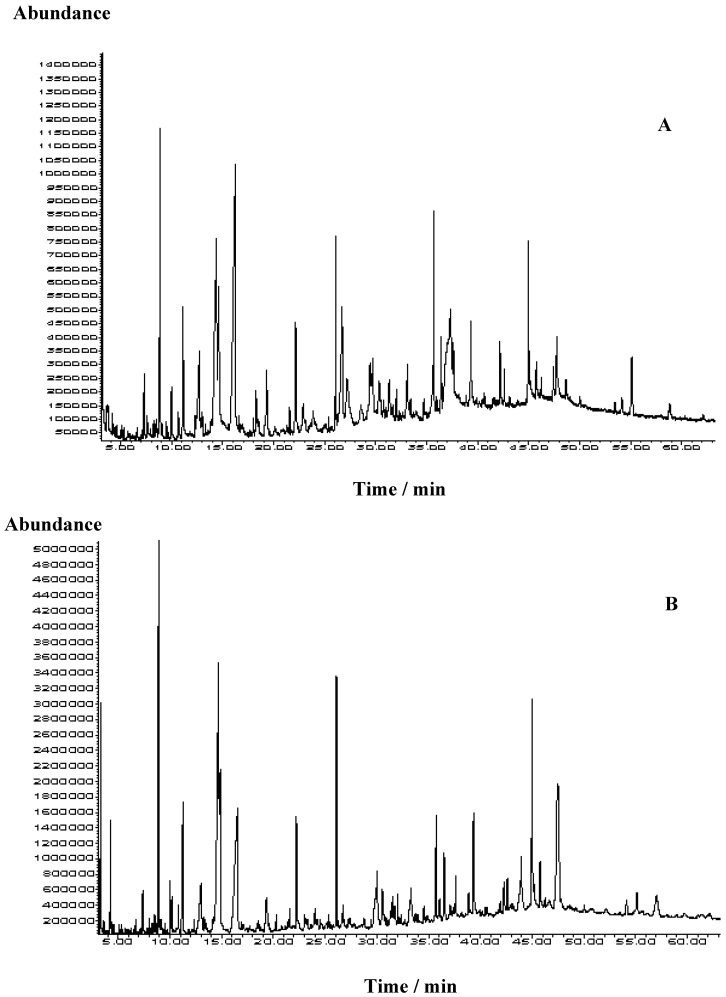
Representative TIC chromatogram of oak honeydew USE extracts of the sample II obtained with the mixture of pentane and diethyl ether 1 : 2, v/v (**A**) and dichloromethane (**B**).

The volatile organic composition results of the samples obtained by ultrasonic assisted extraction (USE) with two solvents followed by GC and GC/MS analysis are presented in Table 2. A representative TIC chromatogram is presented in Figure 1. The most striking difference among the samples was the concentration of 5-hydroxymethylfurfural (5-HMF). Sample I collected in 2005 contained 42.5% of 5-HMF (solvent A) and 64.2% (solvent B), respectively, in contrast to sample II from 2009 with percentages of 4.7% (solvent A) and 6.2% (solvent B). The percentage of 5-HMF in both samples was higher in the more polar solvent dichloromethane. Such a high content was expected since 5-HMF is generated during honeydew storage at room temperature. Prolonged storage of the honeydew led to compositional changes due to caramelization of carbohydrates, Maillard reactions, and decomposition of fructose in relative acid medium of the honeydew [10]. These reactions lead to the formation of HMF or other furan/pyran compounds (such as 2-furanmethanol (1.7%; 0.3%), 5-methylfurfural (1.5%; 0.5%), 1-(2-furyl)-2-hydroxyethanone (5.5%; 4.6%), 2,3-dihydro-3,5-dihydroxy-6-methyl-4H-pyran-4-one (1.3%; 0.3%)). Several furan/pyran derivatives were present in the other sample such as 2-furanmethanol (3.0%; 3.7%), 1-(2-furyl)-2-hydroxyethanone (5.1%; 8.8%), 2,3-dihydro-3,5-dihydroxy-6-methyl-4H-pyran-4-one (2.8%; 4.3%). Although the percentage of 5-HMF predominated in sample I, botanical-origin important compounds were notable, as in sample II.

Shikimic pathway derivatives are of particular interest with respect to the botanical origin of honey since the research focus is oriented toward terpenes, benzene derivatives and norisoprenoids in the honey [2]. The most abundant derivative was phenylacetic acid in sample I (8.1%; 4.7%) and sample II (16.4%; 11.6%) with more pronounced percentages in solvent A. This compound was not found in Spain oak honeydew which may be the consequence of the use of a different extraction technique [2]. Other abundant benzene derivatives were benzoic acid, 4-hydroxybenzoic acid, 4-hydroxybenzyl alcohol, 4-hydroxybenzoic acid, 4-hydroxycinnamic acid and methyl syringate, also not identified in Spain oak honeydew. However, none of the identified benzene derivatives can be proposed as specific markers for oak honeydew since they are present in different honeys.

**Table 2:** Oak honey volatile organic composition isolated by USE.

			Area percentage (%)			
No.	Compound	RI	sample I		sample II	
			A	B	A	B
1.	3-Methylbutanoic acid	< 900	0.1	-	-	-
2.	2-Furanmethanol	< 900	1.7	0.3	3.0	3.7
3.	1,3-Dimethylbenzene^**^	< 900	0.1	-	-	0.2
4.	3-Hydroxy-pentan-2-one	< 900	0.2	-	0.5	-
5.	1-(2-Furanyl)-ethanone	914	0.3	-	-	-
6.	Hexane-2,5-dione	931	0.2	0.1	-	-
7.	5-Methylfurfural	969	1.5	0.5	0.1	0.1
8.	Hexanoic acid^a^	974	0.1	-	-	-
9.	2-Formylpyrrole	1018	0.2	-	0.1	0.4
10.	*p*-Cymene^a^	1031	0.1	-	0.2	-
11.	2-Hydroxy-3-methyl-cyclopent-2-en-1-one	1034	0.3	0.1	-	0.1
12.	Benzyl alcohol^a^	1037	1.2	-	1.4	0.6
13.	Pantoic lactone	1046	-	0.5	-	0.8
14.	Phenylacetaldehyde^a^	1048	0.1	-	-	-
15.	2-Acetylpyrrole	1063	0.2	0.1	0.2	0.1
16.	*trans*-Linalool oxide	1076	0.7	-	0.2	-
17.	4,5-Dimethyl-2-formylfuran	1078	0.4	-	-	-
18.	6-Methyl-2-pyrazinylmethanol	1084	-	0.3	-	0.2
19.	1-(2-Furyl)-2-hydroxyethanone	1087	5.5	4.6	5.1	8.8
20.	*cis*-Linalool oxide	1091	0.4	-	0.2	-
21.	Linalool^a^	1101	0.3	-	0.2	-
22.	2-Phenylethanol^a^	1116	0.7	-	0.9	-
23.	3-Hydroxy-2-methyl-4H-Pyran-4-one (Maltol)	1119	-	0.5	-	1.3
24.	2-Formyl-1-methylpyrrole	1139	1.1	0.5	0.3	0.8
25.	2,3-Dihydro-3,5-dihydroxy-6-methyl-4H-pyran-4-one	1145	1.3	0.3	2.8	4.3
26.	Benzoic acid^a^	1162	4.5	1.8	4.3	3.5
27.	3,5-Dimethylphenol^**^	1181	0.3	0.1	0.4	0.2
28.	3,7-Dimethyl-octa-1,5-dien-3,7-diol	1191	0.4	0.1	0.5	0.8
29.	Dodecane^a^	1200	0.2	-	0.1	0.5
30.	1,2-Benzenediol	1219	0.3	-	0.2	-
31.	2,5-Di(hydroxymethyl)-furan^*^	1229	2.2	-	10.9	17.2
32.	5-Hydroxymethylfurfural	1230	42.5	64.2	4.7	6.2
33.	Phenylacetic acid^a^	1269	8.1	4.7	16.4	11.6
34.	Nonanoic acid^a^	1273	-	-	-	0.1
35.	2-Hydroxybenzoic acid	1311	0.5	0.2	1.6	-
36.	3-Methoxyacetophenone	1321	-	-	0.3	-
37.	3-Hydroxy-4-phenyl-butan-2-one	1354	-	0.5	1.4	0.8
38.	Tetradecane^a^	1400	0.3	0.1	0.4	0.4
39.	4-Hydroxybenzyl alcohol	1426	0.4	0.2	0.6	0.8
40.	Cinnamic acid	1434	0.2	0.2	-	0.2
41.	8-Hydroxyoctanoic acid	1465	0.4	0.3	0.4	0.6
42.	Pentadecane^a^	1500	-	-	0.2	-
43.	4-Methyl-2,6-bis(1,1-dimethylethyl)-phenol	1514	1.4	-	3.6	5.7
44.	4-Hydroxybenzoic acid	1558	1.7	-	2.9	-
45.	Vanillic acid	1566	0.6	0.1	0.4	0.2
46.	Hexadecane	1600	0.8	-	1.5	0.1
47.	3-Oxo-α-ionol	1656	0.3	0.2	-	-
48.	Homovanillic acid	1659	-	-	-	0.5
49.	Syringaldehyde	1662	0.1	0.1	-	0.4
50.	8-Quinolinol	1713	0.2	1.2	-	-
51.	Methyl syringate^a^	1744	1.6	1.2	4.6	3.2
52.	Octadecane^a^	1800	0.1	-	0.2	-
53.	Vomifoliol	1802	1.2	-	1.8	2.4
54.	4-Hydroxycinnamic acid	1817	1.9	-	6.6	-
55.	Hexadecan-1-ol^a^	1882	1.2	0.8	2.0	2.9
56.	Hexadecanoic acid^a^	1963	0.3	0.6	1.7	0.9
57.	(*Z*)-Octadec-9-en-1-ol	2060	2.7	2.8	3.9	8.1
58.	Octadecan-1-ol^a^	2084	0.4	0.6	0.8	1.5
59.	(*Z*)-Octadec-9-enoic acid	2147	-	0.3	2.0	-
60.	Tricosane^a^	2300	0.3	0.3	2.3	2.4
Total identified			91.8	88.4	91.9	92.6

RI = retention indices on HP-5MS column; A = solvent mixture of pentane and diethyl ether (1:2 v/v); B = solvent dichloromethane; - = not identified; ^a^ – identification confirmed with reference compound; ^*^ - tentatively identified; ^**^ - correct isomer not identified.

Another abundant group of identified compounds from Table 2 were aliphatic alcohols, acids and hydrocarbons such as (*Z*)-octadec-9-en-1-ol, hexadecan-1-ol, octadecan-1-ol, hexadecanoic acid, tetradecane, hexadecane, tricosane and others. These compounds can be connected with bees-wax composition [11].

### 2.3. Antiradical and Antioxidant Capacity of the Honeydew and Extracts (DPPH and FRAP assay)

**Figure 2:**
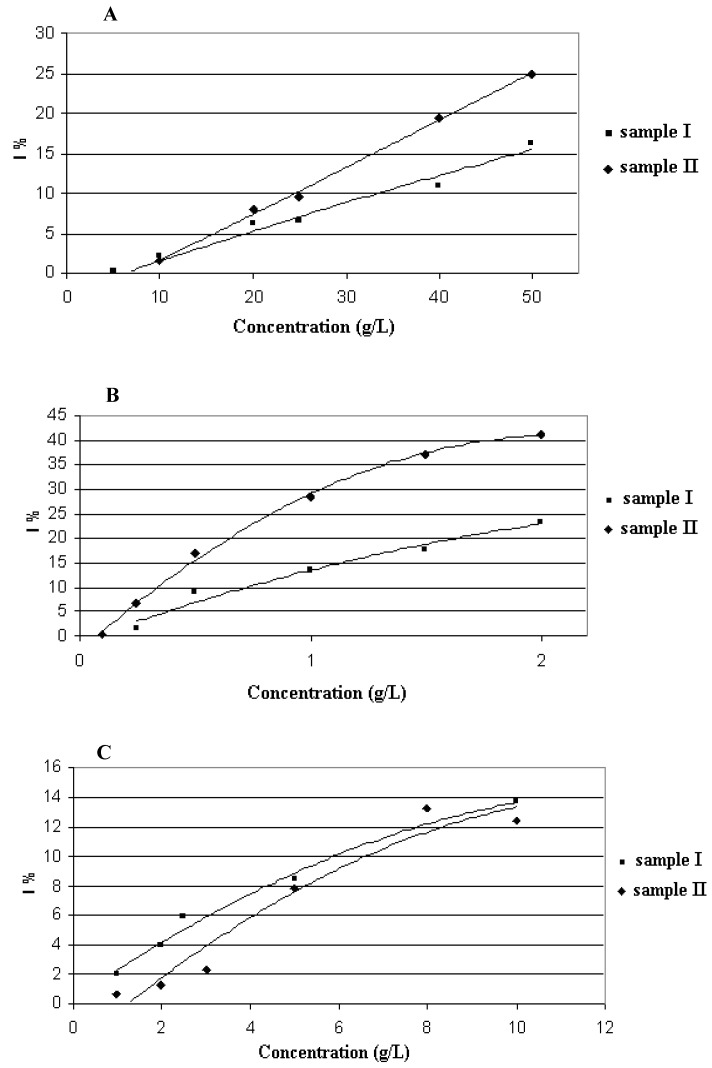
DPPH reduction percentage against increasing concentration of oak honeydew (**A**), pentane - diethyl ether extract (**B**) and dichloromethane extract (**C**).

The antiradical activities (DPPH assay) of oak honeydew and ultrasonic solvent extracts were evaluated from a Trolox solution, Table 1. Antiradical activity was very similar for both oak honeydew samples: 4.5 mmol TEAC/kg (sample I) and 5.1 mmol TEAC/kg (sample II). Therefore it can be concluded that high concentration of 5-HMF in the sample I does not effect antiradical capacity of the honeydew. The percent inhibition of the DPPH radical as a function of the honeydew samples and their ultrasonic solvent extracts concentrations is shown in Figure 2. 

Data from Figure 2 show that when the samples are concentrated, they could saturate DPPH. Also the IC_50_, corresponding to the concentration where 50% of the maximal effect on reduction of DPPH radical, is undetermined because at the maximum concentration of all the samples a maximum of 40% of DPPH inhibition was achieved. These measurements show that the ultrasonic solvent extracts exhibited similar free radical scavenging potential than the honey samples at approximately 4-10 times lower concentration ranges.

**Table 3:** Results of DPPH and FRAP quantitative assay for the honeydew (as mmol TEAC/kg honeydew and mmol Fe^2^/kg honeydew) and the extracts (as mmol TEAC/kg extract or mmol Fe^2^/kg extract). Solvent A and solvent B are described in Table 2.

	sample I		sample II	
	DPPH	FRAP	DPPH	FRAP
	[mmol TEAC/kg]	[mmol Fe^2+^/kg]	[mmol TEAC/kg]	[mmol Fe^2+^/kg]
oak honeydew	4.5	4.8	5.1	16.1
USE extract (solvent A;				
m_IA _= 0.0063 g;	174.3	374.5	256.5	955.9
m_IIA _= 0.0075 g)				
USE extract (solvent B; ;	100.8	127.3	132.1	101.5
m_IB _= 0.0102 g;				
m_IIB _= 0.0167 g))				

m_IA_ - dried extract (solvent A) mass of the sample I; m_IIA_ - dried extract (solvent A) mass of the sample II; m_IB_ - dried extract (solvent B) mass of the sample I; m_IIB_ - dried extract (solvent B) mass of the sample II.

Ultrasonic solvent extract with solvent A (sample I) showed *cca.* 38 times higher antiradical capacity in comparison to the honeydew, while the extract with solvent B (sample I) showed *cca.* 22 times better antiradical activity (Table 3). Antiradical capacity of the extract with solvent A from sample II was 50 times better than oak honeydew capacity, whereas the extract with solvent B (sample II) showed 26 times higher activity. The extracts (with solvent A and B) from sample II exhibited higher antioxidant activity with respect to the same extracts from sample I that can be due to higher 5-HMF concentration in sample I. FRAP antioxidant capacity of honeydew samples ranged from 4.8 to 5.7 mmol Fe^2+^/kg, while extracts with solvent A showed antioxidant activity of 374.5 and 955.9 Fe^2+^/kg, and extracts with solvent B 127.3 and 101.5 mmol Fe^2+^/kg, respectively

## 3. Experimental

### 3.1. Honey Samples

Two oak honeydew samples that were collected in different years were investigated: sample I (2005) and sample II (2009). The samples were obtained from professional beekeepers and no mechanical treatment or heat was used. The combs were placed in the area of wild growing *Quercus frainetto* Ten. Melissopalynological analysis was performed by the methods recommended by the International Commission for Bee Botany [12]. Microscopical examination was carried out on a Hund h 500 (Wetzlar, Germany) light microscope attached to a digital camera (Motic m 1000) and coupled to an image analysis system (Motic Images Plus software) for morphometry of pollen grains. Water content was determined by refractometry, measuring the refractive index, using a standard model Abeé refractometer at 20 ºC. Water content (%) was obtained from the Chataway table [13]. Electrical conductivity was measured in a solution of 20 g honeydew honey in low conductivity water at 20 ºC using conductometer (Hanna HI 8733). All the samples were stored in hermetically closed glass bottles at 4 ºC until the volatiles isolation.

### 3.2. Headspace Solid-Phase Microextraction (HS-SPME)

The isolation of headspace volatiles was performed using a manual SPME fiber with a layer of polydimethylsiloxane/divinylbenzene (PDMS/DVB) obtained from Supelco Co (Bellefonte, PA, USA). The fiber was conditioned prior to use according to the manufacturer’s instructions. For HS-SPME extraction, honey/saturated water solution (5 mL, 1:1 v/v; saturated with NaCl) was placed in a 15 mL glass vial and hermetically sealed with PTFE/silicone septa. The vial was maintained in a water bath at 60 ºC during equilibration (15 min) and extraction (45 min) and was partially submerged so that the liquid phase of the sample was below the water level. All the experiments were performed under constant stirring (1,000 rpm) with a magnetic stirrer. After sampling, the SPME fiber was withdrawn into the needle, removed from the vial, and inserted into the injector (250 ºC) of the GC and GC-MS for 6 min where the extracted volatiles were thermally desorbed directly to the GC column.

### 3.3. Ultrasonic Solvent Extraction (USE)

Ultrasound-assisted solvent extraction (USE) was performed in an ultrasound cleaning bath (Elmasonic Typ S 30 H, Germany) by the mode of indirect sonication (sweep mode), at the frequency of 37 kHz at 25 ± 3 ºC. Forty grams of each sample was dissolved in distilled water (22 mL) in a 100-mL flask. Magnesium sulfate (1.5 g) was added and each sample was extensively vortexed. A mixture of pentane-diethyl ether (1:2, v/v) and dicholoromethane were separately used as the extraction solvent for each honey sample. Sonication was maintained for 30 min. After sonication, the organic layer was separated by centrifugation and filtered over anhydrous MgSO_4_. The aqueous layer was returned to the flask and another batch of the same extraction solvent (20 mL) was added and extracted by ultrasound for 30 min. The organic layer was separated in the same way as the previous one and filtered over anhydrous MgSO_4_, and the aqueous layer was sonicated a third time for 30 min with another batch (20 mL) of the extraction solvent. Joined organic extracts were concentrated to 0.2 mL by distillation with Vigreaux column, and 1 μL was used for GC and GC/MS analyses.

### 3.4. Gas Chromatography and Mass Spectrometry (GC, GC/MS)

Gas chromatography analyses were performed on an Agilent Technologies (Palo Alto, CA, USA) gas chromatograph model 7890A equipped with flame ionization detector, mass selective detector, model 5975C and capillary column HP-5MS ((5%-phenyl)-methylpolysiloxane Agilent J & W GC column, 30 m, 0.25 mm i.d., coating thickness 0.25 μm). Chromatographic conditions were as follows: helium was carrier gas at 1 mL·min^−1^, injector temperature was 250 ºC, and FID detector temperature was 300 ºC. HP-5MS column temperature was programmed at 70 ºC isothermal for 2 min, and then increased to 200 ºC at a rate of 3 ºC·min^−1^ and held isothermal for 18 min. The injected volume was 1 μL and the split ratio was 1:50. MS conditions were: ionization voltage 70 eV; ion source temperature 230 ºC; mass scan range: 30–300 mass units. The analyses were carried out in duplicate.

### 3.5. Data Analysis and Data Evaluation

The individual peaks were identified by comparison of their retention indices (relative to C_9_-C_25_ *n-*alkanes for HP-5MS) to those of authentic samples and literature [14], as well as by comparing their mass spectra with the Wiley 275 MS library (Wiley, New York, NY, USA) and NIST02 (Gaithersburg, MD, USA) mass spectral database. The percentage composition of the samples was computed from the GC peak areas using the normalization method (without correction factors). The component percentages (Table 1 and Table 2) were calculated as mean values from duplicate GC and GC-MS analyses.

### 3.6. Antiradical Activity (DPPH Assay)

The antiradical capacity was determined by the 2,2,diphenyl-1-picrylhydrazyl (DPPH) assay [15]. Oak honeydew samples were diluted first in ultra pure water (1:10, w/v) and then in methanol with different concentrations (g/L) shown in Figure 2. Ultrasonic solvent extracts were carefully evaporated to dryness under nitrogen and dissolved in methanol with different g/L concentrations showed in Figure 2. Spectrophotometric readings were carried out with a UV-Vis Perkin-Elmer Lambda EZ 201 spectrophotometer at 517 nm. DPPH assay was carried out in triplicate for each sample.

The percent of inhibition (I%) of the DPPH radical by the samples was calculated in the following way: I% = [(A_C(0)_ -A_A(t)_)/A_C(0)_] × 100, where A_C(0) _is the absorbance of the control at t = 0min and A_A(t)_ is the absorbance of the samples at t = 60 min. Pure methanol was used to zero the spectrofotometer. The absorbance of DPPH radical without the sample, i.e. the control, was determined.

Quantitative analysis was done using the external standard method (Trolox). A calibration curve in the range of 0.05-1.0 mmol/L was used for Trolox and data were expressed as Trolox equivalent antioxidant capacity (TEAC, mmol/kg). Each sample (50 μL of previously prepared concentration 2 g/L) was dissolved in 2 mL of DPPH 0.04 mmol/L in methanol. The mixtures was shaken and left for 60 min at room temperature in the dark. The absorbance was measured against a control made of 50 µL of methanol and 2 mL of DPPH (the bank was read at t = 0 min and at t = 60 min). 

### 3.7. Total Antioxidant Activity (FRAP Assay)

The ferric reducing-antioxidant assay (FRAP) is based on the reduction at low pH of ferric 2,4,6-tris(2-pyridyl)-1,3,5-triazine [Fe(III)-TPTZ] to the ferrous complex followed by spectrophotometric analysis [15]. The reagent was prepared by mixing 10mM TPTZ with 20mM ferric chloride in acetate buffer (pH 3.6). Quantitative analysis was done using the external standard method (ferrous sulfate, 0.1–2 mmol), correlating the absorbance (λ = 593 nm, UV-Vis Perkin-Elmer Lambda EZ 201) with the concentration. The results were expressed as millimoles per kilogram of Fe^2+^.

## 4. Conclusions

Headspace solid-phase microextraction (HS-SPME) enabled identification of the most volatile organic headspace compounds being dominated by terpenes. The volatile and less-volatile organic composition of the samples was obtained by ultrasonic assisted extraction (USE) with two solvents. The most striking difference among the samples was the concentration of 5-hydroxymethylfurfural (5-HMF). Shikimic pathway derivatives are of particular interest with respect to the botanical origin of honey and the most abundant was phenylacetic acid. Despite the different concentrations of 5-HMF in the samples, the obtained DPPH and FRAP assay values for both samples were quite similar. Ultrasonic solvent extracts showed several dozen times higher antiradical capacity (DPPH assay) in comparison to the honeydew. Antioxidant capacity (FRAP assay) of both extracts were remarkably higher in comparison to honeydew samples. These findings indicate the importance of USE extracts, not just for analytical purposes, but also reveals new potential for further antioxidant and biological activity research. 
